# Promoting Dietary Change Among State Health Employees in Arkansas Through a Worksite Wellness Program: The Healthy Employee Lifestyle Program (HELP)

**Published:** 2009-09-15

**Authors:** Amanda Philyaw Perez, Martha M. Phillips, Carol E. Cornell, Glen Mays, Becky Adams

**Affiliations:** Fay W. Boozman College of Public Health, University of Arkansas for Medical Sciences; University of Arkansas for Medical Sciences, Little Rock, Arkansas; University of Arkansas for Medical Sciences, Little Rock, Arkansas; University of Arkansas for Medical Sciences, Little Rock, Arkansas; Arkansas Department of Health, Little Rock, Arkansas

## Abstract

**Introduction:**

Maintaining a healthy and productive workforce is essential for employers in public and private sectors. Poor nutrition and obesity contribute to chronic diseases and influence health care costs and productivity. Research indicates that eating a healthy diet is associated with lower body mass index and reduced risk for developing chronic disease.

**Methods:**

The Arkansas Department of Health implemented the Healthy Employee Lifestyle Program to encourage wellness among state health employees. During the pilot year, participants completed a health risk assessment at baseline and again after 1 year that assessed diet and physical activity, other health risk factors, and readiness to make behavioral changes. Participants were encouraged to eat healthfully, participate in regular exercise, report health behaviors using a Web-based reporting system, accumulate points for healthy behaviors, and redeem points for incentives. Differences in participants' (n = 214) reported dietary behaviors between baseline and follow-up were assessed using χ^2^ analyses and tests of symmetry.

**Results:**

Consumption of sweets/desserts, fats, protein, grains, processed meats, and dairy did not differ significantly from baseline to follow-up. However, at follow-up more participants reported eating 3 or more fruits and vegetables per day than at baseline and being in the action and maintenance stages of readiness to change for eating 5 or more fruits and vegetables per day and for eating a diet low in fat.

**Conclusion:**

Further study is needed to examine physical activity and other health risk factors to determine whether the program merits a broader dissemination.

## Introduction

Among US adults, only one-third eat enough fruits and a little over one-quarter eat enough vegetables per day to meet nutritional recommendations set in the *Healthy People 2010* national objectives (1), and two-thirds are overweight or obese (2). Poor nutrition and obesity contribute to chronic diseases that result in billions of dollars in medical costs and lost work productivity per year (3-5). Forty-five percent of working-aged Americans have chronic diseases, including hypertension, diabetes, heart disease, stroke, and high cholesterol (6), that are affected by poor nutrition (3) and obesity (7,8). Some research suggests that fruit and vegetable intake is associated with having a lower body mass index (BMI) (9,10) and with reducing the risk for developing chronic disease (10-12). Research suggests that merely increasing the consumption of fruits and vegetables may help with weight management, and increased consumption of high-fiber, energy-poor fruits and vegetables often leads to a spontaneous reduction in fat intake (13). These assumptions about the health and weight management benefits of fruit and vegetable consumption need further investigation.

For decades, state-based programs have promoted public health messages about healthful eating. In recent years, state governments have recognized that their own state workers are as likely as the general public to have poor health habits that are associated with deleterious health outcomes and that directly influence state health care budgets (14). Studies on worksite wellness programs have shown that fruit and vegetable consumption increases when management and peers provide support, create environments that offer healthy choices, and reward participants with incentives (15-17). Consequently, many states have implemented worksite wellness initiatives to improve nutrition, promote physical activity, and reduce obesity among state workers (14,17).

Although states may develop wellness initiatives for state workers, few publish the findings regarding the implementation or evaluation of such programs. Among those few, the North Carolina HeartSmart study showed increased fruit and vegetable consumption among state employees after 1 year of program participation (17). The public workforce size makes program implementation difficult, but states such as North Carolina have initiated approaches (eg, using the Internet) for delivering wellness interventions to reach large numbers of employees (17). Computer or Web-based programs for nutrition promotion make it easier to provide education, monitor dietary intake, and track participant success in a cost-effective way (18). Such programs may provide feasible, affordable solutions to improve employee health in both public and private sectors and thereby reduce the effect of obesity and unhealthy dietary habits on employee health and health care costs.

The Healthy Employee Lifestyle Program (HELP) uses Web-based technology and site-specific program tailoring aimed at decreasing risk for chronic diseases and reducing health care costs among state employees in Arkansas. The Arkansas Department of Health (ADH) developed the HELP intervention and launched a 1-year pilot study in February of 2005. We report the analysis of the nutritional components of the 1-year pilot to assess the effectiveness of the intervention in promoting dietary changes among participants, including shifts in stages of readiness to change dietary practices.

## Methods

### Overview of the Healthy Employee Lifestyle Program

The HELP intervention targeted 10,000 state health and human services employees from 200 county-based offices and 2 central offices in Little Rock, Arkansas. An ADH work team developed HELP in collaboration with the Centers for Disease Control and Prevention (CDC), using resources provided in the *Guide to Community Preventive Services* (*Community Guide*) and incorporating findings from formative research with state employees to assess need and interest in program participation.

The primary goals of HELP were to encourage behavior change in participants, including 1) not smoking or participating in a smoking cessation program, 2) eating 5 or more fruits and vegetables per day, 3) engaging in regular physical activity, 4) achieving or maintaining a healthy BMI (<25.0 kg/m^2^) or participating in a program to reduce BMI, and 5) seeking age-appropriate annual health screenings. Secondary goals targeted behavior change using stages of readiness from the Transtheoretical Model of Behavior Change (19).

All employees in the ADH and Department of Human Services (DHS) were encouraged to participate in HELP and informed about the program via e-mails, newsletters, posters, and other internal communications. Participants enrolled in the program by creating an account through a Web-based system available on the ADH and DHS intranet and by completing a health risk assessment (HRA). The Trale HRA (20) evaluates employee health status, BMI, dietary habits, participation in physical activity, smoking status, stress level, alcohol consumption, and other health risk factors. After completing the HRA, employees received an overall wellness report, including scores that described the person's current state of health, risk factors for diseases and health conditions, and tips to improve health. HELP participants were required to complete an HRA at baseline to be able to enroll in the program and were encouraged to complete follow-up HRAs at approximately 1-year intervals thereafter to assess progress toward personal health goals. This study evaluated the first year of participation and 1 follow-up HRA. In this article, employees who signed up for participation but only completed an initial HRA are referred to as enrollees. Employees who also completed a follow-up HRA are referred to as participants.

Coordinators at the state, regional, and site levels implemented the program by providing coordinator trainings, managing the HELP intervention Web-based system, and distributing materials and information to other coordinators and program participants. Program coordinators periodically transmitted educational information regarding healthful eating, physical activity, state and agency health events, lunch-and-learn sessions, and other health promotion activities.

Enrollees and participants reported their progress through the Web-based system and earned points for self-reported fruit and vegetable consumption, physical activity, smoking cessation, completion of age-appropriate health screenings, weight management, and completion of HRAs. People could post activity in these areas daily, weekly, or monthly. The online system maintained for each enrollee a rolling tally of reward points earned by participants. People could redeem earned points for rewards such as t-shirts, water bottles, and up to 3 days of paid leave. We examined data from the pilot year of the HELP intervention using a cross-sectional cohort design to compare HRA responses to nutritional questions at baseline and approximately 1 year later.

### Study design and participants

A pre-post design with no control group was used because no control group was available for comparison and HRAs were not completed by employees who did not participate in the program. To ensure anonymity, as promised by the program contractors (Trale, Inc, Daleville, Indiana), participant identifiers were not assigned or included in the HRA database. Therefore, HRAs could not be matched to people across time with absolute certainty. Analysis files for the comparison of baseline and follow-up HRAs were created by identifying those dates of birth with more than 1 HRA in the file and with at least 1 HRA completed between February 2005 and March 2006. HRAs were then matched by birth date, age, sex, and height to identify HRAs that were likely completed by the same people. HRAs completed within 8 months of or more than 16 months after the index HRA were excluded from the analysis sets. These strategies generated 2 files (1 for baseline HRAs and 1 for follow-up HRAs), each of which included 214 observations. The University of Arkansas for Medical Sciences institutional review board reviewed and approved this study.

### Study measures

The HRA assessed intake frequency of fat, sweets/desserts, fruits, vegetables, protein, grains, dairy, processed meats, and fried foods by using categorical response options (never, 1-4 times/wk, 5-7 times/wk, 2 times/d, ≥3 times/d) for each category. Stage of readiness to change (ie, precontemplation, contemplation, preparation, action, maintenance) (19) was assessed for eating a low-fat diet, taking daily steps to achieve or maintain a healthy weight, and eating 5 or more fruits and vegetables daily. Response options for these 3 questions were categorical: "not doing this and have no plans to start" (precontemplation), "plans to do this within the next 6 months" (contemplation), "plans to do this within the next 30 days" (preparation), "started doing this within the last 6 months" (action), and "have been doing this for at least 6 months or more" (maintenance). Because of the small sample size, the stages of readiness to change were collapsed into 3 categorical variables of precontemplation/contemplation, preparation, and action/maintenance.

### Statistical analysis

Data were analyzed using SAS version 9.1 (SAS Institute, Inc, Cary, North Carolina). Univariate analyses were completed to describe program enrollees at baseline. A pre-post analysis of HRA responses for the matched sample was completed using the Bowker test of symmetry and the McNemar χ^2^ test. The null hypothesis was no difference in distribution of responses in the baseline and follow-up groups (α = .05).

## Results

Of the 10,000 ADH and DHS employees, 10% (n = 1,017) enrolled in HELP during the first year, February 2005 to March 2006. Most enrollees and participants were female and white (Table 1). Mean BMI of enrollees and participants was 30 kg/m^2^, and approximately 75% of HELP enrollees and participants were either overweight or obese.

At follow-up, more participants ate 3 or more servings of vegetables per day than they did at baseline (26.2% vs 13.6%, *P* = .03) (Table 2). The data showed an overall trend of increased fruit consumption between baseline and follow-up. There was a shift in consumption of 3 or more fruits per day from 10.8% at baseline to 17.3% at follow-up (*P =* .08). Participants' consumption of the more healthful food groups of proteins, grains, and dairy did not increase significantly between baseline and follow-up. No significant differences toward decreased consumption of sweets/desserts, fats, fried foods, and processed meats were observed.

Participants progressed between baseline and follow-up in stages of readiness to change for eating 5 or more fruits and vegetables per day (*P* = .002) and for eating a low-fat diet (*P* = .04) (Table 3). For eating 5 or more fruits and vegetables per day, 42% of participants were in the preparation stage and 41% were in the action or maintenance stages. At follow-up, the percentage of participants in the preparation stage fell to 27% while the percentage in the action or maintenance stage increased to 59% (*P* = .002). Similarly, for eating a diet low in fat, 29% of participants were in the preparation stage and 49% were in the action or maintenance stage at baseline; at follow-up, the percentage of participants in the preparation stage fell to 21% and the percentage in the action or maintenance stage increased to 59% (*P* = .04).

## Discussion

Our findings from the 1-year pilot of the HELP program suggest that a Web-based worksite wellness incentive program may improve nutritional health behaviors of public-sector employees. The HELP program encourages behavior change through 3 main approaches: 1) providing an overall wellness report with tips for improving health, 2) rewarding health behaviors with points redeemable for incentives, and 3) providing education and peer support. These pilot results are promising, given the small sample of 214 participants. A larger sample would have been helpful in detecting more modest changes in behavior.

Fruit and vegetable consumption was the only dietary behavior rewarded by the HELP intervention. Other desirable behaviors (eg, decreasing consumption of fats or sweets) were not directly rewarded by the program. This reward system may have contributed to the lack of significant change in other dietary behaviors. Further investigation is necessary to determine how best to achieve change in the broader range of dietary behaviors.

Our findings are consistent with other wellness interventions that reported increased fruit and vegetable consumption and decreased fat consumption among program participants (16,17). For example, both the Seattle and Treatwell 5-A-Day worksite wellness studies reported increased fruit and vegetable consumption among participants (21,22). Similarly, the Worksite Internet Nutrition program reported decreased fat consumption and increased fruit and vegetable consumption among participants by using applied nutritional behavior change principles through an e-mailed intervention (23).

The changes observed among HELP participants may not be solely due to program participation. During the period of pilot implementation, Arkansas reformed state policies and organizational structures responsible for health programs in Arkansas, and media outlets heralded the state's multiple efforts to improve health by promoting nutrition and physical activity. Longer-term longitudinal studies are needed to determine the HELP program's effectiveness.

A limitation of this study was the inability to match precisely the baseline and follow-up HRAs for participants. Although the cross-sectional samples were matched closely, the interpretation of findings of change over time would have been strengthened by a true longitudinal sample. This study is limited further by the nature of the data, which were self-reported. The influence of this limitation may be mitigated somewhat by the failure of the program to reward change over time; therefore, there was little or no incentive for participants to skew their responses on the follow-up HRA in any single direction. Having 1 year between baseline and follow-up HRAs may have minimized the self-report bias because participants were not likely to remember previous responses. Further, to the extent that people in the matched samples were different from people for whom no match was possible, our ability to generalize these findings to program participants overall is limited. People who remained in the program and were motivated to complete a follow-up HRA could have been systematically different from those who failed to do so, further limiting the generalizability of these findings.

People with a high BMI tend to use health services more often, which lowers their work productivity because of absence and contributes to higher insurance premiums for employers (4,5,24,25). Most (75%) HELP enrollees reported BMIs in the overweight or obese ranges, suggesting that HELP was able to reach and recruit the desired, higher-risk employees. Our data do not include information on health care use or days missed from work, both of which would be indicators of program effectiveness.

The HELP pilot program produced positive outcomes in a brief period. Results suggest that the HELP intervention can be a feasible, affordable, easily implemented health behavior intervention that shows some promise for improving dietary behaviors of working adults. Findings suggest that, in time, risk, morbidity, and mortality may decrease if participants continue to increase healthy behaviors and decrease less healthy behaviors (5). Increased fruit and vegetable consumption is an easily communicated health message that shows promise for decreasing risk for chronic disease (17,21,22). Further analysis of the HELP data for physical activity and other health risk factors will be examined to determine whether the program merits a broader dissemination.

## Figures and Tables

**Table 1:** Selected Demographic Characteristics of Enrollees and 1-Year Follow-up Matched Participants, Healthy Employee Lifestyle Program (HELP), Arkansas, 2005-2006

Characteristic	Enrollees (n = 1,017)^a^	Matched Sample Participants (n = 214)^b^
**Age, y, %**		
20-44	46	47
45-64	53	52
≥65	1	1
**Sex, %**		
Male	12	8
Female	88	92
**Race/ethnicity, %**		
Black	22	18
White	75	78
Latino	1	1
Other	2	3
**BMI, kg/m^2^**		
Mean (SD)	30.5 (7.5)	30.4 (7.3)
Obese^c^	45	47
Overweight^d^	29	29

Abbreviations: BMI, body mass index; SD, standard deviation.

a Enrollees signed up for HELP and completed a baseline health risk assessment.

b Matched sample participants represent the enrollees identified by birth date, age, sex, and height with a completed health risk assessment at baseline and follow-up.

c BMI ≥30.0 kg/m^2^.

d BMI 25.0-29.9 kg/m^2^.

**Table 2:** Self-Reported Nutritional Consumption, at Baseline and Follow-Up, Participants of the Healthy Employee Lifestyle Program (HELP), Arkansas, 2005-2006

**How often do you eat or drink . . . ?**	% at Baseline	% at Follow-Up	*P* Valueª
**Vegetables**			
Never	0.5	0.5	.03
1-4 times/wk	21.0	17.3	
5-7 times/wk	32.2	32.2	
2 times/d	32.7	23.8	
≥3 times/d	13.6	26.2	
**Fruits**			
Never	3.7	3.3	.08
1-4 times/wk	37.4	28.0	
5-7 times/wk	26.2	28.0	
2 times/d	22.0	23.4	
≥3 times/d	10.8	17.3	
**Proteins (chicken, red meat, pork, beans, nuts)**			
Never	0	0	.10
1-4 times/wk	14.5	17.8	
5-7 times/wk	36.0	43.0	
2 times/d	37.9	32.7	
≥3 times/d	11.7	6.5	
**Grains (pasta, rice, bread)**			
Never	1.4	0.9	.81
1-4 times/wk	32.7	34.1	
5-7 times/wk	31.3	33.6	
2 times/d	19.2	20.1	
≥3 times/d	15.4	11.2	
**Dairy (milk, cheese, yogurt)**			
Never	0.9	0	.82
1-4 times/wk	32.7	31.3	
5-7 times/wk	31.8	37.9	
2 times/d	24.8	21.5	
≥3 times/d	9.8	9.4	
**Sweets/desserts**			
Never	3.7	3.3	.75
1-4 times/wk	58.9	65.0	
5-7 times/wk	27.1	23.8	
2 times/d	7.0	4.7	
≥3 times/d	3.3	3.3	
**Fat (cream sauces, butter)**			
Never	3.7	5.1	.75
1-4 times/wk	71.5	68.2	
5-7 times/wk	15.4	18.2	
2 times/d	7.0	6.1	
≥3 times/d	2.3	2.3	
**Processed meats (hot dogs, lunch meats)**			
Never	17.3	17.3	.85
1-4 times/wk	71.0	73.8	
5-7 times/wk	10.3	7.5	
2 times/d	1.4	0.9	
≥3 times/d	0	0.5	
**Fried foods**			
Never	8.9	9.8	.16
1-4 times/wk	72.0	71.5	
5-7 times/wk	16.8	13.1	
2 times/d	2.3	3.7	
≥3 times/d	0	1.9	

a χ^2^ test used for unadjusted comparisons between baseline and 1-year follow-up, *P* < .05.

**Table 3:** Percentage of Participants in Stages of Change Model From Baseline to Follow-up, by Health Behavior, Healthy Employee Lifestyle Program (HELP), Arkansas, 2005-2006

Health Behavior	Stage of Change^a^, %			*P* Value^b^
	Precontemplation/Contemplation	Preparation	Action/Maintenance	
**Eating ≥5 fruits and vegetables per day**				
Baseline	16.4	42.5	41.1	.002
Follow-up	14.0	26.6	59.4	
**Eating a low-fat diet**				
Baseline	22.4	29.0	48.6	.04
Follow-up	20.1	21.0	58.9	
**Taking daily steps to achieve or maintain a healthy weight**				
Baseline	6.1	21.5	72.4	.69
Follow-up	5.6	17.8	76.6	

a See Methods section for description of stages.

b χ^2^ test used for unadjusted comparisons between baseline and 1-year follow up, *P* < .05.
